# A LuALS Mutation with High Sulfonylurea Herbicide Resistance in *Linum usitatissimum* L.

**DOI:** 10.3390/ijms24032820

**Published:** 2023-02-01

**Authors:** Caiyue Liu, Tianbao Zhang, Xinsen Yang, Liu Wang, Yan Long, Agula Hasi, Xinwu Pei

**Affiliations:** 1 Biotechnology Research Institute, Chinese Academy of Agricultural Sciences, Beijing 100081, China; 2 Key Laboratory of Herbage & Endemic Crop Biology, Ministry of Education, School of Life Sciences, Inner Mongolia University, Hohhot 010070, China

**Keywords:** acetolactate synthase, herbicide resistance, flax, mutant

## Abstract

The cultivation of herbicide-resistant crops is an effective tool for weed management in agriculture. Weed control in flax (*Linum usitatissimum* L.) remains challenging due to the lack of available herbicide-resistant cultivars. In this study, a mutant resistant to acetolactate synthase (ALS)-inhibiting herbicides was obtained by ethyl methanesulphonate (EMS) mutagenesis using an elite cultivar, Longya10. Whole-plant dose–response assays revealed that, compared to Longya10, the mutant was 11.57-fold more resistant to tribenuron-methyl (TBM) and slightly resistant to imazethapyr (resistance index (mutant/Longya10) < 3). In vitro acetolactate synthase assays showed that the relative resistance of the mutant was 12.63 times more than that of Longya10. A biochemical analysis indicated that there was a Pro197Ser (relative to the *Arabidopsis thaliana* ALS sequence) substitution within the LuALS1, conferring high resistance to sulfonylurea herbicides in the mutant. Additionally, two cleaved amplified polymorphic sequence (CAPS) markers, *Bsa*I-*LuALS1* and *Eco*O109I-*LuALS1*, were developed based on the mutation site for marker assistant selection in breeding. Moreover, the mutant did not cause losses in natural field conditions. We find a mutant with ALS-inhibiting herbicide resistance chemically induced by EMS mutagenesis, providing a valuable germplasm for breeding herbicide-resistant flax varieties.

## 1. Introduction

Weeds influence crop yield and quality by competing with crops for water, light, soil nutrients, and physical space [1]. Generally, weeds are the main global threat to crop production, reducing the yield potential of major crops by 34%, higher than insect pests (18%) and diseases (16%) [2]. Chemical control of weeds is an effective method in modern agricultural production. Acetolactate synthase (ALS) is a key enzyme for the synthesis of branched-chain amino acids, including leucine, valine, and isoleucine, and ALS-targeting inhibiting herbicides are favored due to their broad herbicidal spectrum, low use, high selectivity to crops, and low toxicity to mammals [3,4,5]. Upon binding to ALS, herbicides block substrate access to the enzyme active channel and inhibit enzyme activity, thereby disrupting protein synthesis and causing loss of greenness, yellowing, and eventually death of the plant [6]. According to chemical structures, the ALS-inhibiting herbicides are classified into six categories: pyrimidinyl benzoates (PYB), triazolopyrimidine-type 1/type 2 (TP), sulfonylureas (SU), imidazolinones (IMI), triazolinones (TZ), and sulfonanilides [7]. SU herbicides, as the first commercially applied ALS-inhibiting herbicides, are widely used in some crops such as wheat, rice, corn, soybean, and rape [5].

In recent years, different methods including natural mutations, chemical mutagenesis, and gene editing techniques have been used to obtain ALS herbicide-resistant crops. The sunflower inbred line RW-8 was obtained by crossing a cultivated sunflower line with resistant wild sunflower. RW-8 was highly resistant to SU and IMI herbicides due to the W574L mutation in Ahasl1 [8]. By mutagenizing the indica rice variety HHZ with ethyl methanesulphonate (EMS), a herbicide resistant mutant KH-9 was obtained. It was found that the bases at positions 1642 and 1643 were converted from TG to AT, resulting in a W574M amino acid substitution. Another rice line, JTD-001, with high IMI herbicide resistance was obtained by backcrossing [9]. By performing EMS mutagenesis on a rapeseed cultivar N131, a mutant, M342, resistant to 90 g a.i.ha^−1^ thifensulfuron or 360 g a.i.ha^−1^ imazethapyr was developed, and sequencing analysis revealed that the BnAHAS3 sequence in this mutant had a W574L replacement, which made the mutant acquire a high level of SU and IMI herbicide resistance [10]. By editing the *ALS1* gene in soybean using the CRISPR/Cas9 system, a chlorsulfuron-resistant line, ALS1-18, with a Pro178Ser mutation was generated [11]. After introducing a CT-nCas9 editing vector into a maize inbred line ZC01, a double edited mutant for both *ZmALS1* and *ZmALS2* genes was created, which was able to tolerate 15 times the recommended field dose of chlorsulfuron [12]. Zhang et al. targeted the TaALS-Pro174 locus in wheat by using the pnCas9 editing system and obtained a homozygous mutant with six editing alleles in T_2_ generation, which showed high levels of resistance to nicosulfuron and metsulfuron [13]. These results suggest that it is feasible to endow crops with herbicide resistance through the mutagenesis of the *ALS* genes that results in amino acid substitution.

As a self-pollinating diploid oil crop, flax (*Linum usitatissimum* L.) is widely planted in Asia, America, and Europe, with 4.14 million hectares planted worldwide in 2021 [14]. Linseed oil is rich in essential A-linolenic acid (ALA) at 55%, which is known to reduce the incidence of cardiovascular and other chronic diseases [15,16]. In addition to being used for making food and animal feed, flax is also used to produce industrial products such as paint and flax flooring [17]. However, dense planting has led to many weeds, especially broad-leaved weeds, which has reduced the yield of flax by 30% [18]. At present, only a few herbicides are available for broad leaf weed control in flax fields, including amidosulfuron, bentazone, bromoxynil+clopyralid, and iodosulfuron [19]. Long-term use of a single herbicide results in weed resistance, such as *Xanthium strumarium* L. and *Liriodendron chinense* (Hemsl. Sargent), which increases the difficulty of weed control [18]. Therefore, it is necessary to explore resistance genes and develop new germplasms of flax resistant to ALS-inhibiting herbicides for effective control of broad-leaved weeds.

However, little research has been reported on the resistance of flax to ALS-inhibiting herbicides. Mchughen et al. transformed the *Arabidopsis thaliana ALS* gene carrying the mutation into flax hypocotyl tissue and obtained 14 transgenic lines with chlorsulfuron resistance at the 20 g ha^−1^ level [20,21]. The resulting linseed variety, CDC Triffid, was registered in Canada and approved for use in food, feed, and commercial plantations [22]. However, it was deregistered in 2001 due to GM safety regulations, and the export of Canadian flaxseed was suspended [17]. To date, CDC Triffid is the only transgenic flax cultivar reported to be resistant to SU herbicides. In addition, according to the online database of the China Pesticide Information Network (http://www.chinapesticide.org.cn/, accessed on 12 September 2021), the use of chlorsulfuron has been banned in China since 2015. Therefore, it is urgent to cultivate ALS-resistant flax germplasm resources. Beside this, there are not any reports about ALS-inhibiting herbicide resistance varieties developing in flax.

In this study, we first used EMS to treat one of the wide planting flax varieties, Longya10, in China to construct a mutant library. Based on herbicide resistance screening experiments, we selected one SU herbicide resistant mutant. Then, we explained the resistance mechanism of the mutant through ALS enzyme activity, sequencing, and protein structure analysis. Finally, two cleaved amplified polymorphic sequence (CAPS) markers were developed for rapid and accurate identification of the mutation sites of the resistance gene. Taken together, our findings provided not only valuable germplasm for herbicide-resistant breeding and weed control in flax, but also useful molecular makers for helping to quickly identify useful resources in flax.

## 2. Results

### 2.1. Development of Tribenuron-methyl (TBM)-Tolerant Mutant R10

To construct an EMS mutant library of flax, 5 kg of seeds of Longya10 (M_0_) were treated with a 0.9% EMS mutagenesis solution. A total of 50–60 individual plants per row after thinning were planted, and the M_1_ population contained 100,000 individual plants. At seed maturity stage, about 90,000 individual plants were harvested in a mix. The flax seedlings in the M_2_ generation were sprayed with 9.28 g a.i.ha^−1^ tribenuron-methyl (TBM), which is half of the recommended concentration (RC). Three weeks later, 27 surviving plants were screened out, and 13 of these were finally harvested. Among the 13 lines of M_3_ generation, one line, R10, showed higher resistance under the same TBM treatment. Then, each M_3_ plant of R10 was harvested separately. The M_4_ plants derived from mutant R10 all showed normal growth after 2 × RC TBM and were chosen for further study. In addition, R10 did not cause yield reduction under natural conditions and exhibited precocity and lodging resistance (Table S1).

### 2.2. Mutant R10 Showed High TBM-Resistance in the Greenhouse

**Figure 1:**
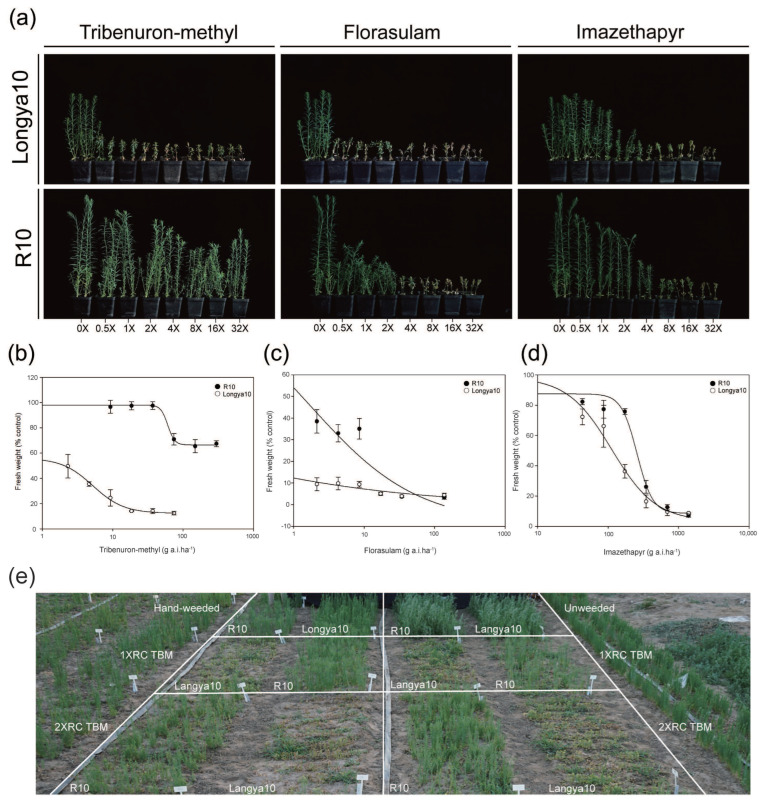
Herbicide resistance test of mutant R10 in greenhouse and field conditions. (**a**) Resistance analysis of R10 to three types of acetolactate synthase (ALS)-inhibiting herbicides. Tribenuron-methyl (TBM) (SU), florasulam (TP), and imazethapyr (IMI) were sprayed at eight concentrations of 0, 1/2, 1, 2, 4, 8, 16, and 32 × RC (recommended concentration). (**b**) Dose–response curves of R10 and Longya10 to TBM. (**c**) Dose–response curves of R10 and Longya10 to florasulam. (**d**) Dose–response curves of R10 and Longya10 to imazethapyr. (**e**) Field phenotypes of R10 and Longya10 sprayed with TBM (1–2 × RC) for 21 d, including hand-weeded and unweeded controls.

The resistance levels of R10 to TBM, imazethapyr, and florasulam were detected through whole-plant dose–response assays. Longya10 plants died after treatment at all application rates of TBM, and mutant R10 exhibited resistance to 1 × RC TBM. When the herbicide concentration was increased to 8 × RC TBM, R10 presented growth inhibition. However, at higher dosages of 32 × RC TBM, R10 retained a survival rate of 100% (Figure 1a). The GR50 (herbicide effective index causing the plant growth reduction of 50%) value of R10 was 60.53 g a.i.ha^−1^, a dose which corresponds to a 3× application rate under field conditions. The resistance was 11.57 times higher than that of Longya10 (5.23 g a.i.ha^−1^) (Figure 1b and Table 1). Compared with Longya10, mutant R10 plants survived under 1 × RC florasulam, but their growth was severely inhibited (Figure 1a). The GR50 value of R10 was much lower than the recommended doses for field application (1 × RC) (Figure 1c and Table 1). Apparently, R10 was sensitive to florasulam. All seedlings of R10 and Longya10 survived under 1 × RC imazethapyr without symptoms of chlorosis or necrosis (Figure 1a). The GR50 value of Longya10 was 112.40 g a.i.ha^−1^, which was higher than the field recommended dose (1 × RC). The resistance index (RI) of the mutant R10 was 2.29, indicating that R10 did not have a significant resistance to imazethapyr (Figure 1d and Table 1). These results indicated R10 was very highly resistant to TBM.

### 2.3. Mutant R10 Exhibited Sustained TBM-Resistance in the Field

To determine the resistance of R10 to TBM and the weed control effect of the herbicide, we sprayed 1 and 2 × RC TBM to the tested plants and set up both hand-weeded and unweeded controls in the field. After TBM treatment for 21 d, R10 maintained a survival rate of 100% in both spraying dose treatments, whereas the plants of Longya10 all died (Figure 1e). Our survey found that in R10 planting plots, weed types were mainly *Chenopodium album* L., *Portulaca oleracea* L., *Amaranthus retroflexus* L., and *Eleusine indica* (L.) Gaertn. These broad-leaved weeds are sensitive to TBM, with weed control close to 100% under 1 × RC TBM treatment. Therefore, R10 could be potentially used with TBM for weed control management in flax fields.

### 2.4. The TBM Resistance in Mutant R10 Was Mediated by ALS Insensitivity

**Figure 2:**
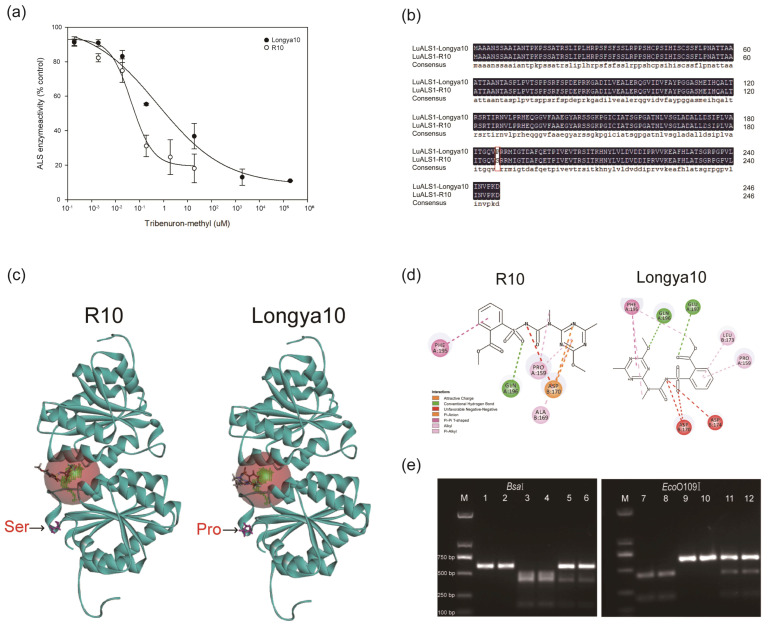
ALS enzyme activity, coding protein sequences, protein structural, and molecular marker identification of *LuALS1* in mutant R10 and its wild-type Longya10. (**a**) The enzyme activity reaction curve of R10 and Longya10. (**b**) LuALS1 protein sequence alignment of mutant R10 and Longya10. The amino acid at 186 (AtALS-P197) is marked in red. (**c**) Homology model of LuALS1 docking with TBM in R10 and Longya10. (**d**) A 2D view of the LuALS1-TBM docking model in R10 and Longya10. (**e**) Two CAPS markers of *Bsa*I-*LuALS1* and *Eco*O109I-*LuALS1*. M, 2k marker; 1/7, Longya10; 2/8, Macbeth; 3/4, 9/10, R10; 5/11, F1 (R10 × Longya10); 6/12, F1 (Macbeth × R10).

ALS activity inhibition kinetics are often used to verify whether the ALS-inhibiting herbicide resistance in mutants is based on the target mutation [23]. The log-logistic model accurately plotted the in vitro inhibition curves of ALS activity at different concentrations of TBM for R10 and Longya10 (Figure 2a). For R10, the concentration of TBM required to achieve 50% inhibition of ALS activity was about 0.581 μM, much higher than the 0.046 μM for Longya10. The relative resistance for R10 was 12.63 times higher than that of Longya10. This result suggested that the TBM resistance of R10 was based on the target site.

### 2.5. Pro197Ser in LuALS1 Conferred Resistance to Mutant R10

To analyze the genetic behavior of the mutation, R10 was crossed with two susceptible varieties, Longya10 and Macbeth. After 1 × RC TBM treatment, three phenotypes of tolerant (R, asymptomatic), intermediate (I, apical part of leaf curl, no death), and susceptible (S, growth arrest, apical yellowing, death) were observed in the F_2_ populations (Figure S1). The segregation ratio of herbicide resistance was 1:2:1 in two F2 populations tested by χ^2^ goodness of fit (Table 2). This indicated that the resistance trait in R10 was caused by a mutation in a semi-dominant nuclear coding gene.

Using the local BLAST method, 11 putative *LuALS* gene members of the ALS family in the flax genome were identified. The genes were named in the order from *LuALS1* to *LuALS11* (Table 3). To determine the key amino acid mutant sites in R10, all 11 putative *LuALS* genes were amplified from 10 plants each with R, I, and S phenotypes from the F_2_ population. Sequence alignment with Longya10 revealed that different sense mutations occurred in *LuALS* genes of F2 plants with different phenotypes. However, the same mutation site, C556T, existed in *LuALS1* gene of all plants with R and I phenotypes, resulting in the conversion of proline to serine at position 186 (AtALS-P197) (Table 3 and Figure 2b). Additionally, this locus was homozygous in R10, heterozygous in F_1_ plants, and unmutated in Longya10. These results indicated that TBM resistance in R10 was caused by a base substitution in the *LuALS1* gene.

### 2.6. LuALS1 Protein Structure Was Influenced by Pro197Ser

The resistance mutations to ALS-inhibiting herbicides are concentrated in eight amino acids, including A122, P197, A205, D376, R377, W574, S653, and G654. These are located in the substrate access channel or catalytic pocket of ALS and are direct contact points with the herbicides [3]. Therefore, LuALS1-P186, the site homologous to AtALS-P197, is expected to confer tolerance to SU herbicides with the conversion of proline to serine. To understand the molecular basis of the herbicide resistance in R10, we constructed a homology model of LuALS1 docking with TBM based on the crystal structure of AtALS (Figure 2c). The 2D view of the LuALS1–TBM docking model showed that the main forces between TBM and LuALS1 were conventional hydrogen bonds, Pi-Alkyl and Pi-Pi T-shaped. TBM formed hydrogen bonds with Gln196 and Glu of LuALS1; Pi-Alkyl interacted with Phe195, Leu173, and Pro159; and Pi-Pi T-shaped interacted with Phe195. In contrast, the main interaction between TBM and LuALS1 decreased after Pro197Ser replacement. Only a hydrogen bond was formed at Gln196, the Pi-Alkyl interaction occurred at Pro159 and Ala169, and similarly, the Pi-Pi T-shaped interaction occurred at Phe195, increasing the Pi-anion interaction at Asp170 (Figure 2d). The reduction in hydrogen bonds and the weakening of hydrophobic forces resulted in the TBM no longer entering the active channel in a nearly orthogonal manner with two rings, thus acquiring resistance to TBM in R10 (Figure S2).

### 2.7. Two CAPS Markers Were Developed for Mutant R10 Genotyping

In R10, the nucleotide sequence GGTCCC, located at 552–557 bp from the translation start site, was mutated to GGTCTC. Sequence GGTCCC can be recognized by restriction enzyme *Eco*O109I, while the corresponding GGTCTC in R10 cannot be recognized. Additionally, sequence GGTCTC in R10 can be recognized by the restriction enzyme *Bsa*I, while the corresponding GGTCCC cannot be recognized. Thus, two CAPS markers *Bsa*I-*LuALS1* and *Eco*O109I-*LuALS1* were developed to detect the resistance mutation site in *LuALS1*. PCR analysis of the DNAs from the plants cleaved by *Bsa*I showed two fragments of 405 bp and 177 bp for R10; three fragments of 582 bp, 405 bp, and 177 bp for F_1_; and a complete fragment of 582 bp for Longya10 and Macbeth. In contrast to these results, when the DNAs from the plants were digested by *Eco*O109I R10, they presented a complete fragment of 582 bp; the F_1_ plants presented three fragments of 582 bp, 399 bp, and 183 bp; and Longya10 and Macbeth presented two fragments of 399 bp and 183 bp (Figure 2e). The genotypes identified by the CAPS markers were consistent with those identified by sequencing.

To detect this new herbicide-resistant locus in flax, *LuALS1* allele sequences from these susceptible varieties, including Tianya2, Zhangya2, Baya11, Baxuan3, Mengya1, Ningya19, and Jinya7, were analyzed in the main planting areas of Gansu, Hebei, Inner Mongolia, Ningxia, and Shanxi provinces. In these susceptible species, the nucleotide sequence 552–557 bp from the translation start site was GGTCCC, which was identical to the unmutated sequence (Figure S3). These results suggested that the two CAPS markers *Bsa*I-*LuALS1* and *Eco*O109I-*LuALS1* could effectively detect the *LuALS1* genotype with wide applicability.

## 3. Discussion

The production and application of herbicide-resistant mutants are valuable for weed control in flax fields. Prior to our study, the only herbicide-resistant flax was created by transferring a mutated *ALS* gene from *Arabidopsis thaliana* [20]. In this study, we created an ALS-inhibiting herbicide-resistant mutant R10 by EMS mutagenesis. R10 showed a strong resistance to SUs and survived a 32 × RC TBM treatment with no symptoms of yellowing or necrosis (Figure 1a). A dose of 1 × RC TBM was used for field weed control experiments with good results. It is obvious that R10 can avoid the symptoms of herbicide poisoning caused by improper or uneven use of herbicide concentrations. In addition, the short residual period of TBM in the soil provides farmers with more options for crop rotation [5].

The biochemical mechanisms that confer ALS-inhibiting herbicide resistance in plants are divided into target sites and non-target sites [24]. The target resistance can be interpreted by the relative tolerance levels of the ALS enzyme [25]. Results of ALS enzyme activity assays in this study showed that R10 was 12.63-foldresistant to TBM (Figure 2a), which suggested that this resistance was associated with the mutation sites within the *ALS* genes. Genetic sequence analysis of R10 revealed a base substitution, C→T, at 556 from the translation start site in *LuALS1* gene, resulting in the replacement of proline by serine at amino acid position 186 (AtALS-P197) (Figure 2b). This mutation has been reported as the basis of ALS resistance in several species, such as soybean [26], rape [27], maize [12], and sugar beet [28]. In these studies, Pro197Ser always conferred high resistance to SU and cross-resistance to TP, but little resistance to IMI. This is consistent with our findings. Additionally, previous studies have shown that Pro197 is one of the most common mutation sites in ALS protein for resistance to ALS-inhibiting herbicides. When it was replaced by Ala, Arg, Asn, Gln, His, Ile, Leu, Lys, Met, Ser, Thr, and Trp, plants developed high resistance to SU herbicides and Pro197Leu also conferred resistance to IMI herbicides [29,30,31,32]. Notably, although R10 obtained resistance to florasulam (TP) compared to Longya10, plant growth was severely inhibited with 0.5 × RC florasulam, and its GR50 value was lower than the recommended dose in the field (Figure 1c and Table 1). Therefore, TP is not suitable for weed control in flax fields.

Moreover, two CAPS markers, *Bsa*I-*LuALS1* and *Eco*O109I-*LuALS1*, were developed to detect the mutation site C556T in the *LuALS1* gene. These two methods can accurately identify the genotype and are applicable to several main cultivars. Our results provide two new co-dominant molecular markers for the assisted breeding of flax varieties using R10 as a parent. Finally, it was found that R10 did not suffer a yield penalty and showed precocity and lodging resistance (Table S1). The reduction in the plant height and growth period of R10 may be caused by background mutations, which is a potential research area that needs to be explored in the future.

In summary, this is the first report on herbicide resistance induced by mutation of endogenous *ALS* genes in flax. The resistance phenotype presented here and the biochemical and molecular characteristics of LuALS1 suggest that R10 is expected to provide a powerful tool for the control of broad-leaved weeds in flax fields.

## 4. Materials and Methods

### 4.1. Seed Mutagenesis and Resistant Mutants Screening

A flax variety Longya10 was kindly provided by the Institute of Crop Research, Gansu Academy of Agricultural Sciences. The flax seeds of 5 kg weight were soaked in a 0.9% EMS solution at 25 °C for 18 h. The treated M_1_ generation seeds were sown in a field under conventional management and the seeds were mixed harvested at maturity. The M_2_ generation seeds were sown in the field and sprayed with 0.5 × RC TBM at the 8–10 leaves stage [21]. After the 21 d test period, the resistant phenotype was observed and the plants with normal growth were marked as candidate plants. The M_3_ generation planted and sprayed with the same concentration of TBM (0.5 × RC) at the 8–10 leaves stage for rescreening. The resistant strains from the rescreening were harvested singly. Resistance isolation tests were carried out on progeny using 2 × RC TBM. The material selected were then investigated for yield and quality traits, including 1000-grain weight, yield per plant, oil content, the proportion of the five main linseed fatty acids, etc. The oil content and the proportion of the five main linseed fatty acids were determined using an infrared analyzer DA7200 (Gansu Academy of Agricultural Sciences). These materials were self-pollinated and planted in the field trial station of the Chinese Academy of Agricultural Sciences in Langfang city, Hebei province, China (39°35′28″ N, 116°35′53″ E).

### 4.2. Herbicide Tolerance Tests in the Greenhouse and Field

Firstly, the mutants were sown in pots filled with a soil mixture (soil/vermiculite/perlite = 2:1:1, *v*/*v*/*v*) and cultured under standard greenhouse conditions (23 °C, 16 h light/8 h dark, 60–70% humidity) for herbicide resistance tests. Three categories of ALS-inhibiting herbicides were selected: TBM (SU) (Shengbang Greenland Chemical Co., Ltd.; Jinan, China, 1 × RC 18.56 g a.i.ha^−1^), imazethapyr (IMI) (Cynda Chemical Co., Ltd.; Jinan, China, 1 × RC 86.62 g a.i.ha^−1^), and florasulam (TP) (Luba Chemical Co., Ltd.; Jinan, China, 1 × RC 4.28 g a.i.ha^−1^). Seedlings with 8–10 leaves were treated with different concentrations of herbicides: TBM (0, 1/128, 1/32, 1/8, 1/4, 1/2, 1, 2, 4, 8, 16, and 32 × RC for Longya10 and 0, 1/2, 1, 2, 4, 8, 16, and 32 × RC for the mutant), imazethapyr, and florasulam (0, 1/2, 1, 2, 4, 8, 16, and 32 × RC for Longya10 and mutant). Each herbicide dose included three pots of seedlings (four seedlings in each pot) and repeated three times.

The fresh weight of aboveground plant parts was measured after herbicide spraying for 21 d. The GR50 and RI were calculated by SigmaPlot software (v.14.0, SigmaPlot Software, Chicago, IL, USA) using a log-logistic model: y = C + (D − C)/[1 + (x/GR_50_ or I_50_) ^b^], in which x stands for the herbicide dose, y for the percentage of fresh weight, C and D for the minimum and maximum values of inhibition rate, respectively, and b for the slope. RI = GR50_(mutant)_/GR50_(Longya10)_. In this study, when RI ≥ 10, 5 ≤ RI < 10, and 2 ≤ RI < 5, the level of resistance was considered as high, moderate, and low resistance, respectively. The mutants were also sown in the field for the herbicide resistance test in May 2022. Each 1 × 3 m plot contained ten rows and the rows were spaced 30 cm apart. Each row contained 50–60 seedlings. Three rates of TBM were used: 0×, 1×, and 2 × RC. Two control plots were set up for hand-weeded and unweeded. Treatments of 1× and 2 × RC were tested in duplicate. The survival rates of R10 and Longya10 were recorded after the spraying for 21 d.

### 4.3. ALS Enzyme Extraction and Activity Assay

Seedlings at the 8–10 leaves stage were used for in vitro assays of ALS enzyme activity. The fresh leaves of Longya10 and the mutant was harvested and snap-frozen in liquid nitrogen. The protocol for ALS extraction and assay was that according to Yu et al. [33]. TBM concentrations were set at 1.897 × 10^−4^, 1.897 × 10^−3^, 1.897 × 10^−2^, 1.897 × 10^−1^, 1.897, 18.97, 1.897 × 10^3^, and 1.897 × 10^5^ μM. The assay was repeated three times with independent extractions. I50 (the herbicide concentration which inhibits the ALS activity by 50%) and RI were calculated using SigmaPlot 14.0. The statistical analysis method was the same as before (y means the percentage of enzyme activity), RI = I50_(mutant)_/I50_(Longya10)_.

### 4.4. Genetic Behavior Analysis of the Mutated Trait

Two F2 populations were obtained by crossing mutant R10 with Longya10 and Macbeth, respectively. The F2 population was planted in a greenhouse and sprayed with 1 × RC TBM. Three weeks later, three phenotypes, tolerant (R, asymptomatic), intermediate (I, apical part of leaf curl, no death), and susceptible (S, growth arrest, apical yellowing, death), were investigated and the segregation rate of the target gene in the F2 population was tested by χ2 goodness of fit to determine the genetic characteristics in the mutant.

### 4.5. Amplification of the LuALS Genes

The ALS protein sequence of *Arabidopsis thaliana* was retrieved from the database TAIR (The Arabidopsis Information Resource, https://www.arabidopsis.org/) and then aligned with the protein sequences of flax obtained from the genomic database Phytozome (https://phytozome.jgi.doe.gov/pz/portal.html#!info?alias=Org_Lusitatissimum, accessed on 24 December 2021). Genomic DNA was extracted from 10 plants showing different phenotypes of herbicide resistance (R, I, and S). Specific primers were designed according to sequence differences (Table S2). PCR was performed using a high fidelity DNA polymerase P525-AA (Vazyme Biotech., Nanjing, China). The amplification procedure started from a pre-denaturation at 95 °C for 5 min, then 35 cycles of denaturation at 95 °C for 30 s, annealing at 56 °C for 30 s, and finally an extension at 72 °C for 45 s. The products with correct size were sequenced (Tsingke Biotechnology Co., Ltd. China). Sequence alignment was performed using DNAMAN (V9.0) to identify the mutation sites.

### 4.6. Protein Structure Prediction

The LuALS1 sequences in Longya10 and the mutants were submitted to SwissModel (https://swissmodel.expasy.org/interactive, accessed on 6 May 2022) for homology modeling based on the structural model of the complexes formed by AtALS and chlorpyrifosulfuron [34]. The small molecule ligands of TBM were downloaded from the PDB Protein database (https://www.rcsb.org/, accessed on 6 May 2022) [6]. Protein–ligand docking analysis was performed using Discovery Studio 2019.

### 4.7. Development of Molecular Markers

A pair of primers were designed to specifically amplify the *LuALS1* gene: *Bsa*I/*Eco*O109I-*LuALS1*-F: 5′-TTCCTTCCCAATGCTACCAC-3′ and *Bsa*I/*Eco*O109I-LuALS1-R: 5’-CTTAGGAACGTTAATCAACACAGGG-3′. The PCR amplification was programmed by the procedure of pre-denaturation at 95 °C for 5 min, 35 cycles of denaturation at 95 °C for 30 s, annealing at 56.5 °C for 30 s, and extension at 72 °C for 45 s. The purified PCR products were digested with the restriction endonuclease in a 50 μL reaction system consisting of 1 μg PCR product, 10 U endonuclease, 5 μL 10 × buffer, and distilled water. The reaction program was performed at 37 °C for 60 min followed by 65 °C for 20 min. The digested products were detected in a 1.5% agarose gel after electrophoresis.

## Figures and Tables

**Table 1:** Determination of the resistance levels to ALS-inhibiting herbicides of mutant R10.

Family	Herbicides	Field Dose (g a.i.ha^−1^)	GR50 (g a.i.ha^−1^) ^a^		Resistance Index ^b^
			R10	Longya10	R10/Longya10
SU	Tribenuron-methyl	18.56	60.53 ± 9.63	5.23 ± 0.35	11.57
TP	Florasulam	4.28	1.75 ± 1.46	<1	>1.75
IMI	Imazethapyr	86.62	257.46 ± 43.21	112.40 ± 22.75	2.29

Note: ^a^ GR50: herbicide concentration that caused a 50% reduction in plant growth. ^b^ RI (resistance index) = GR50 of R10/GR50 of Longya10.

**Table 2:** Inheritance analysis on TBM resistance in mutant R10.

Cross	Tested Plants					χ^2^ (1:2:1)	*p* Value
	Population	Total	R ^a^	I ^b^	S ^c^		
R10 × Longya10	F_2_	203	40	103	60	3.985	0.136
Macbeth × R10	F_2_	90	26	37	27	2.867	0.239

Note: ^a^ Tolerant (R), ^b^ Intermediate (I), ^c^ Susceptible (S). The trait segregation ratio was tested by χ^2^ goodness of fit.

**Table 3:** Sense mutation information of the 11 putative *LuALS* genes in different F_2_ plants.

Code	Gene ID	Nucleotide Position	Base Change	Amino Acid Change	Phenotypes ^a^
LuALS1	Lus10022445	556	C→T	Pro_186_Ser	R, I
LuALS2	Lus10022446	239	G→C	Gly_80_Ala	R, I, S
		243	G→C	Met_81_Ile	R, I, S
		276	A→C	Glu_92_Asp	R, I, S
		295	G→C	Ala_99_Glu	R, I, S
LuALS3	Lus10016751	539	T→G	Val_180_Gly	S
		538, 539	GT→TG	Val_180_Cys	I, S
LuALS4	Lus10032040	241	A→T	Thr_81_Ser	R, I, S
LuALS5	Lus10032037	250	A→T	Thr_84_Ser	R, I, S
		445	G→C	Glu_149_Gln	R, I, S
LuALS6	Lus10032041	302	A→G	Gln_101_Arg	R, I, S
LuALS7	Lus10025595	211	A→T	Thr_211_Ser	R, I, S
		354	C→G	Asn_118_Lys	R, I, S
		407	A→G	Lys_136_Arg	R, I, S
		409	C→A	Gln_137_Lys	R, I, S
		1091	C→G	Pro_364_Arg	R, S
		1253	C→T	Ala_418_Val	R, S
		1339	C→A	Leu_447_Ile	R, S
LuALS8	Lus10035359	221	T→A	Val_74_Glu	R, I, S
LuALS9	Lus10029955	544	G→A	Ala_182_Thr	R, I, S
		1246	T→G	Leu_416_Val	R, I, S
LuALS10	Lus10027061	- ^b^	-	-	-
LuALS11	Lus10035207	- ^b^	-	-	-

Note: ^a^ This mutation occurred in plants with the following phenotypes: tolerant (R), intermediate (I), and susceptible (S). ^b^ There was no sense mutation in this gene.

## Data Availability

Data are contained within the article and the Supplementary Materials.

## Supplementary Materials

The following supporting information can be downloaded at: https://www.mdpi.com/article/10.3390/ijms24032820/s1.
