# Supplementary material for: A LuALS Mutation with High Sulfonylurea Herbicide Resistance in Linum usitatissimum L
Source: Int J Mol Sci. 2023 Feb 1;24(3):2820. doi: 10.3390/ijms24032820 (PMC9917167; doi:10.3390/ijms24032820)
Supplement: Supplementary file 1 [file ijms-24-02820-s001.zip › Legends for Supplementary Figures.pdf]

Figure S1. Three response phenotypes of R, I and S appeared in the two F2 populations (R10×Longya10 and Macbeth×R10) after the treatment of spraying 1×RC TBM.

Figure S2. Hydrophobic forces of LuALS1 in mutant R10 and wide type Longya10.

Figure S3. *LuALS1* sequence alignment in several flax cultivars of Longya10, Baya11, Jinya7, Mengya1, Ningya19, Tianya2, Zhangya2, Baxuan3 and Macbeth. The nucleotide at position 556 was marked in red.
