# Supplementary material for: A LuALS Mutation with High Sulfonylurea Herbicide Resistance in Linum usitatissimum L
Source: Int J Mol Sci. 2023 Feb 1;24(3):2820. doi: 10.3390/ijms24032820 (PMC9917167; doi:10.3390/ijms24032820)
Supplement: Supplementary file 1 [file ijms-24-02820-s001.zip › Table S1, Table S2.pdf]

Table S1 Comparison of yield traits between mutant R10 and Longya10.

| | Growth period (day) | Plant height (cm) | Technological length (cm) | Number of stems per plant | Number of branchs per plant | Number of effective capsules per plant | Number of seeds per capsule | 1000-grain weight (g) | yield per plant (g) | Oil content (%) | Linolenic acid (%) | Linoleic acid (%) | Oleic acid (%) | Stearic acid (%) | Palmitic acid (%) |
|---|---|---|---|---|---|---|---|---|---|---|---|---|---|---|---|
| Longya10 | 105 | 68.32±0.79 | 40.55±1.95 | 3.38±0.33 | 7.45±0.30 | 44.09±1.17 | 8.04±0.16 | 6.47±0.11 | 2.30±0.13 | 36.38±0.51 | 47.58±2.45 | 11.84±0.15 | 28.21±1.50 | 6.59±0.67 | 5.75±0.27 |
| R10 | 90 | 49.32±2.53 | 24.38±1.50 | 2.98±0.16 | 7.58±0.20 | 41.91±1.40 | 8.25±0.30 | 6.46±0.09 | 2.23±0.03 | 35.22±0.06 | 51.44±0.49 | 12.32±0.18 | 25.35±0.59 | 5.99±0.29 | 5.61±0.07 |
| *p* value | - | <0.01 | <0.01 | 0.13 | 0.54 | 0.11 | 0.34 | 0.88 | 0.45 | 0.02 | 0.06 | 0.02 | 0.04 | 0.23 | 0.41 |

Note: The data represent means and standard deviations (STD) of three replicates. Statistically significant differences were determined by two-tailed paired Student's t-tests.

Table S2 Primers used for amplification by PCR of *LuALS* genes.

| Primer or probe | Sequence (5′→3′) | Use |
|---|---|---|
| Lus10022445-F1 | ATGGCCGCCGCTAATTC | Gene cloning |
| Lus10022445-R1 | CTTAGGAACGTTAATCAACACAGGG | Gene cloning |
| Lus10022446-F1 | TCCTAAAAATCCTTGCAGGC | Gene cloning |
| Lus10022446-R1 | GTACTTGATTCTCCCATCTCCC | Gene cloning |
| Lus10025595-F1 | CCCTTCCTCCATTTCCACT | Gene cloning |
| Lus10025595-R1 | TGTCAAAATCCCCCGTCAG | Gene cloning |
| Lus10027061-F1 | CTCTTCACCTTCCATCGCT | Gene cloning |
| Lus10027061-R1 | TATATCAAGAACGTCACGTCGG | Gene cloning |
| Lus10029955-F1 | ATGGCGACGATCCCTTTC | Gene cloning |
| Lus10029955-R1 | CTTCACCGGCAAATTCTCGAC | Gene cloning |
| Lus10032040-F1 | TTCTTCTCCAAACAGACCTCCC | Gene cloning |
| Lus10032040-R1 | CTCCACGATCGGAGTTTCC | Gene cloning |
| Lus10032041-F1 | GATCACGAGCTTTCCCTTCAT | Gene cloning |
| Lus10032041-R1 | TTAAAAGCTCCGCCGCT | Gene cloning |
| Lus10032037-F1 | ATGAATGTTCAAGAGCTCGC | Gene cloning |
| Lus10032037-R1 | TCAGGGAATTCGCCAAGGA | Gene cloning |
| Lus10035207-F1 | TATCGTCGCCATCACGGGA | Gene cloning |
| Lus10035207-R1 | AGCATCCTTAAAAGCCCCAC | Gene cloning |
| Lus10035359-F1 | ATGAATGTTCAGGAGCTGGC | Gene cloning |
| Lus10035359-R1 | GAACATGTTCTTGATGAGGCAC | Gene cloning |
| Lus10016751-F1 | ATGGCCGCTAATTCCTCC | Gene cloning |
| Lus10016751-R1 | CTAGTACTCGCTTCTCCCATC | Gene cloning |
| LuALS1-35S-F | agaacacgggggactgaattcATGGCCGCCGCTAATTCC | Plasmid construction |
| LuALS1-35S-R | tgcggccgcctcgagcccgggCTAAATATCCTTAGGAACGTTAATCAACA | Plasmid construction |
| *Bsa*I/*Eco*O109I-LuALS1-F | TTCCTTCCCAATGCTACCAC | CAPS[a] |
| *Bsa*I/*Eco*O109I-LuALS1-R | CTTAGGAACGTTAATCAACACAGGG | CAPS |

Note: [a] CAPS, cleaved amplified polymorphic sequence.
