# Supplementary figures and images for: A LuALS Mutation with High Sulfonylurea Herbicide Resistance in Linum usitatissimum L
Source: Int J Mol Sci. 2023 Feb 1;24(3):2820. doi: 10.3390/ijms24032820 (PMC9917167; doi:10.3390/ijms24032820)

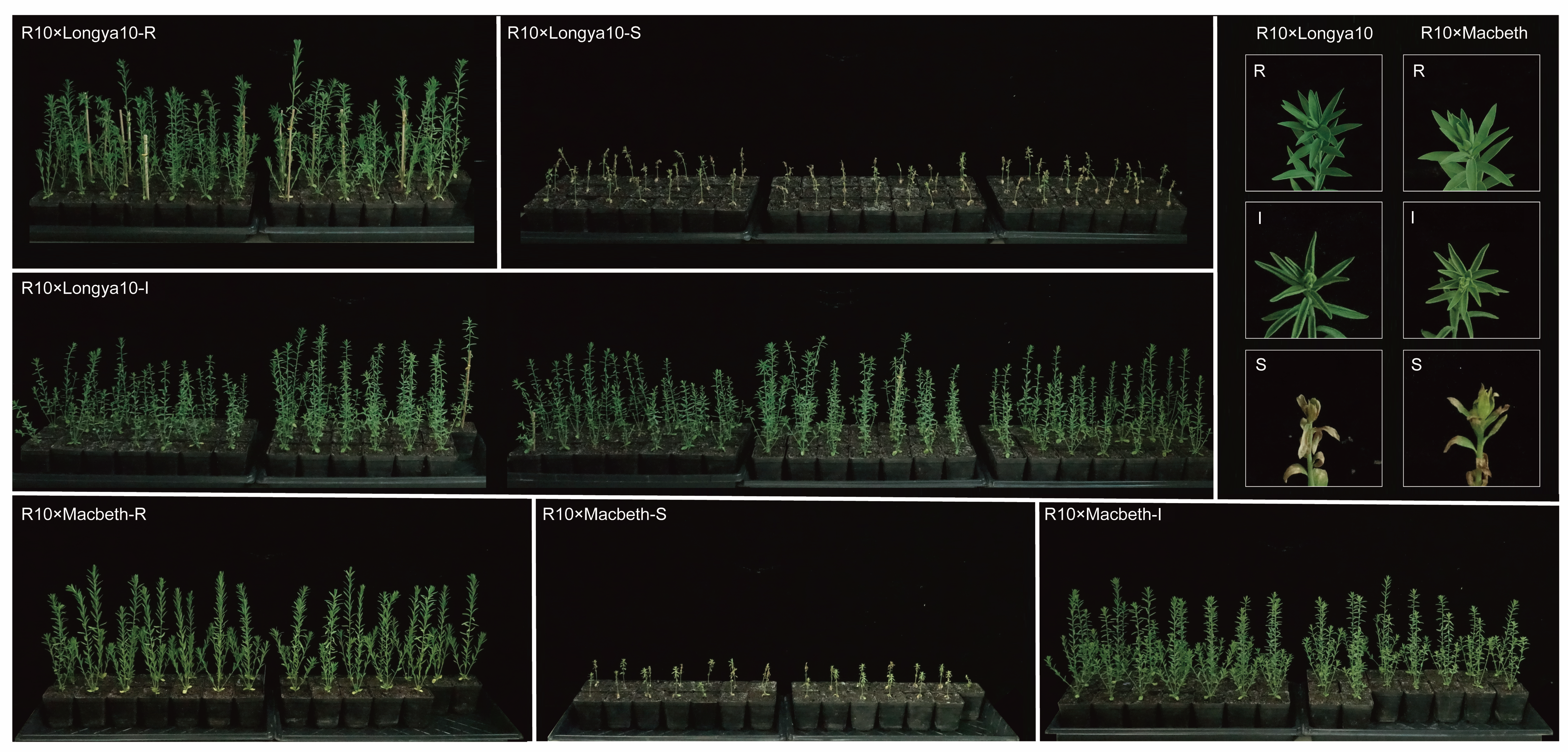

Supplement: Supplementary file 1 [file ijms-24-02820-s001.zip › Figure S1.tif]

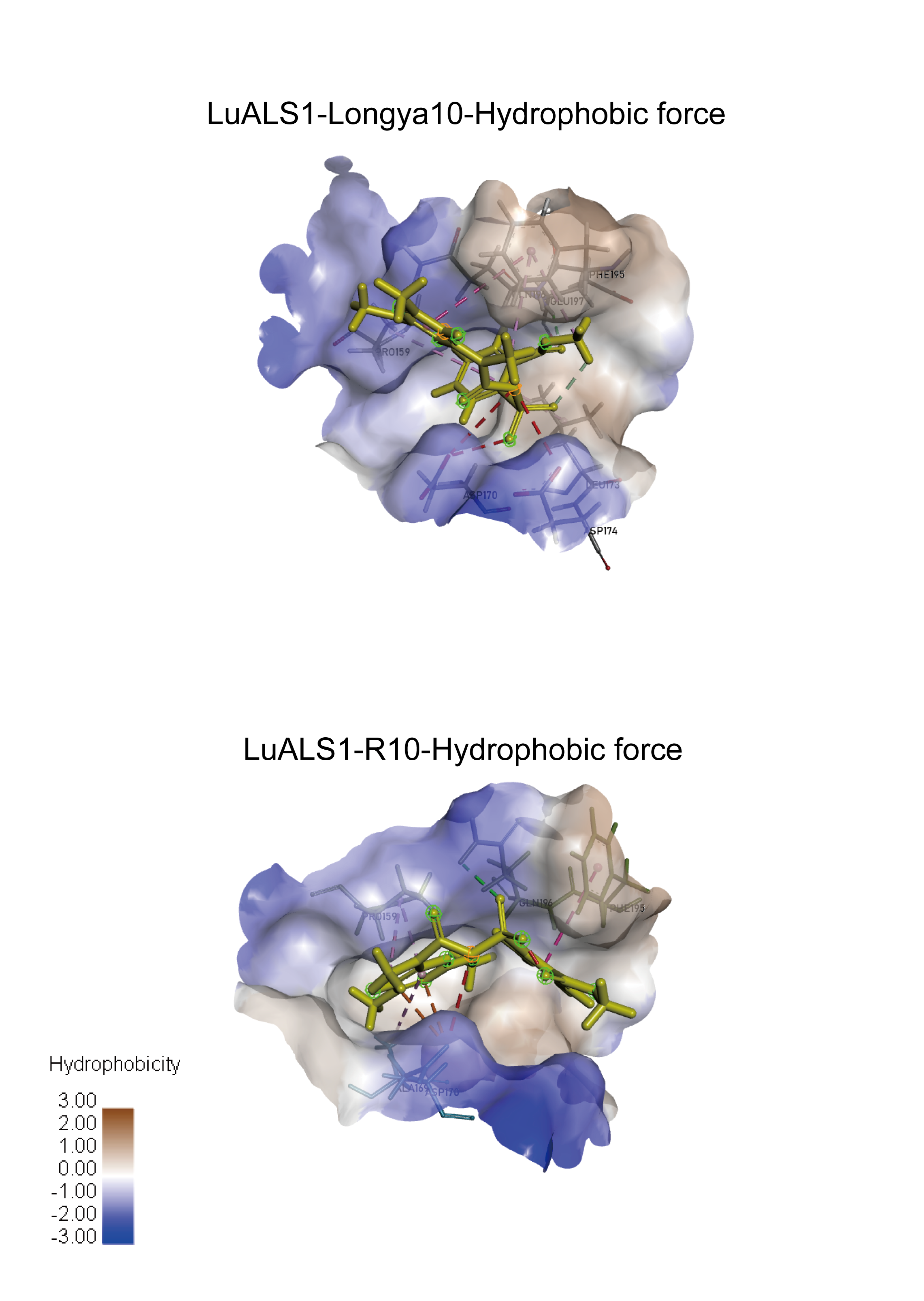

Supplement: Supplementary file 1 [file ijms-24-02820-s001.zip › Figure S2.tif]

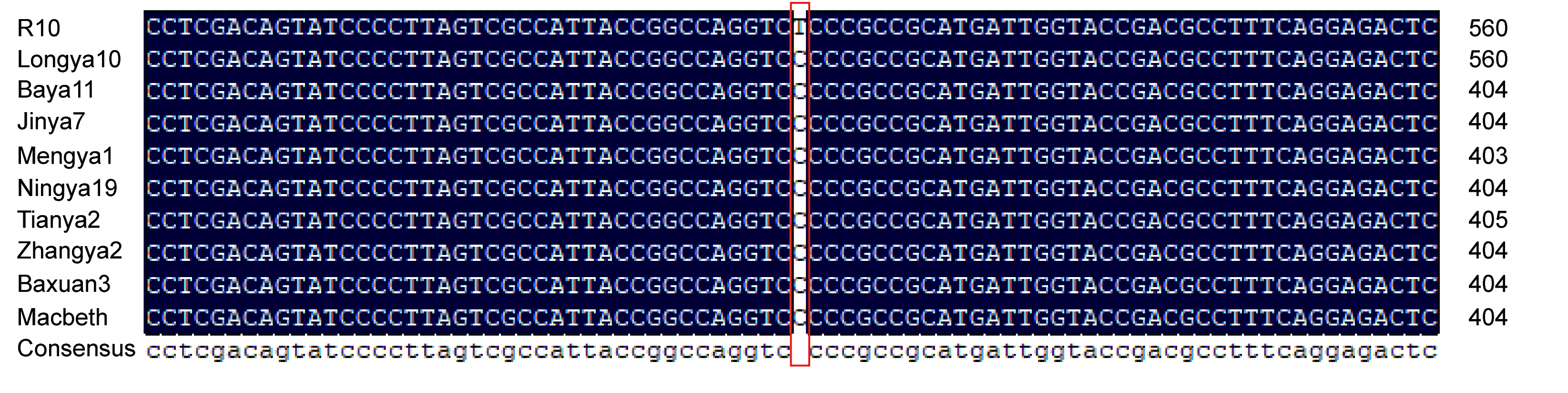

Supplement: Supplementary file 1 [file ijms-24-02820-s001.zip › Figure S3.tif]
